# Deep Learning for Multi‐Disease OCT Classification in Clinical Retina Practice Using Attention Mechanisms and Contrastive Learning

**DOI:** 10.1049/htl2.70106

**Published:** 2026-10-25

**Authors:** Khosro Rezaee, Safoura Farsi, Hossein Ghayoumi Zadeh

**Affiliations:** ^1^ Department of Biomedical Engineering Meybod University Meybod Iran; ^2^ Department of Biomedical Engineering Amirkabir University of Technology (AUT) Tehran Iran; ^3^ Department of Electrical Engineering Faculty of Engineering Vali‐e‐Asr University of Rafsanjan Rafsanjan Iran

**Keywords:** artificial intelligence, deep learning, diabetic retinopathy, optical coherence tomography, retinal diseases

## Abstract

Artificial intelligence (AI) is increasingly used in ophthalmic imaging to support early detection of vision‐threatening retinal diseases such as age‐related macular degeneration, diabetic retinopathy (DR) and choroidal neovascularisation (CNV). However, optical coherence tomography (OCT)‐based deep learning models may remain sensitive to image degradation, struggle to resolve subtle inter‐class morphological differences and show reduced reliability beyond controlled benchmark settings. We propose AESNet‐SEC, a lightweight attention‐enhanced SqueezeNet framework integrating multi‐scale dilated convolutions, spatial attention, squeeze‐and‐excitation recalibration and contrastive representation learning. The framework was evaluated on the Retinal OCT‐C8 dataset (24,000 images; eight classes) and a prospectively collected clinical dataset from Zeyaei Hospital, Iran (9732 OCT B‐scans; four classes). AESNet‐SEC achieved 98.56% accuracy on OCT‐C8 and 96.33%–96.98% across four repeated clinical evaluations. Ablation and ROC analyses showed the strongest overall discrimination for the complete configuration, while controlled Gaussian‐noise testing revealed greater degradation for DR and drusen. Grad‐CAM visualisations highlighted anatomically plausible retinal regions, with independent retinal‐specialist review providing qualitative support for the observed activation patterns. Overall, AESNet‐SEC combines strong multi‐class discrimination, computational efficiency, controlled robustness and visual interpretability, while broader patient‐disjoint, multi‐centre validation remains necessary.

## Graphical abstract

We developed AESNet‐SEC, a lightweight optical coherence tomography (OCT) classification framework that integrates multi‐scale dilated convolutions, spatial attention, squeeze‐and‐excitation recalibration and contrastive learning for multi‐disease retinal diagnosis. Evaluated on the eight‐class OCT‐C8 benchmark and a prospectively collected clinical cohort, AESNet‐SEC achieved 98.56% accuracy on OCT‐C8 and 96.33%–96.98% across repeated clinical evaluations, while Grad‐CAM analysis provided anatomically plausible visual explanations. These findings support AESNet‐SEC as a computationally efficient and interpretable framework for OCT‐based retinal disease classification, although broader patient‐disjoint, multi‐centre validation remains necessary.

## 1 Introduction

The retina is a highly organised neural tissue that converts incident light into neural signals and supports the transmission of visual information to the brain [1]. Disruption of retinal structure or function can result in substantial and, in some conditions, irreversible visual impairment. Retinal disorders such as age‐related macular degeneration (AMD), diabetic retinopathy (DR), diabetic macular oedema (DME), choroidal neovascularisation (CNV) and central serous retinopathy (CSR) therefore require timely recognition to facilitate appropriate clinical management [2].

Imaging techniques such as optical coherence tomography (OCT) and fundus photography have become indispensable for diagnosing and monitoring retinal diseases. Among these modalities, OCT has assumed a particularly important role because it provides rapid, non‐invasive, high‐resolution cross‐sectional visualisation of retinal microstructure and enables detailed assessment of disease‐related anatomical alterations [3]. Despite OCT's widespread clinical use, interpretation remains reliant on trained ophthalmologists and retinal specialists. This dependence can become particularly challenging in high‐volume screening environments and healthcare settings with limited access to retinal expertise. Automated OCT analysis therefore has potential value as a decision‐support approach for prioritising examinations, recognising suspicious structural patterns and supporting more consistent assessment without replacing specialist judgment.

Deep learning has substantially expanded the capabilities of automated OCT analysis by enabling hierarchical feature representations to be learned directly from retinal images. Convolutional neural networks (CNNs), transfer‐learning strategies, lightweight architectures and multi‐scale feature extraction have demonstrated strong performance across retinal classification tasks [4, 5, 6, 7]. Nevertheless, improvements in classification accuracy must be considered together with computational requirements, representation quality and the ability to discriminate between disease categories with partially overlapping OCT appearances.

Recent studies have increasingly addressed this balance between predictive performance and computational efficiency. Saha et al. [8] developed a lightweight CNN‐based OCT classification framework incorporating efficient convolution, global‐local feature fusion, and squeeze‐and‐excitation (SE) recalibration, demonstrating that compact models can retain competitive performance across multiple OCT benchmarks. In parallel, Khriss et al. [9] showed that OCT classification performance can be materially affected by hyperparameter selection and demonstrated the value of systematic optimisation and component‐level ablation rather than relying on manually selected configurations alone. These findings reinforce the importance of evaluating architectural components and training settings explicitly when developing efficient retinal classification systems.

A separate challenge concerns generalisability. High performance on a curated benchmark does not necessarily imply equivalent behaviour under routine clinical acquisition. Differences in imaging quality, device characteristics, disease prevalence, patient populations and disease severity can produce distribution shifts between development and clinical data [3]. More broadly, recent multi‐disease retinal‐image studies have emphasised that model compactness should be evaluated alongside cross‐dataset reliability rather than benchmark performance alone [10]. This distinction is particularly important for multi‐class retinal classification, where visually related disease categories may share structural characteristics and where errors may become concentrated among diagnostically subtle classes.

Several complementary learning strategies have been proposed to address these limitations. Data augmentation can broaden the effective diversity of the training distribution, whereas transfer learning can provide informative initialisation when domain‐specific labelled data are limited [11]. Contrastive learning offers a different form of representation optimisation by encouraging related image representations to remain close while increasing separation between diagnostically distinct samples. Importantly, contrastive learning should be viewed as a representation‐learning mechanism rather than as a direct solution to class imbalance. Evidence from retinal OCT imaging has shown that contrastive pretraining can improve discrimination when clinically relevant differences are subtle and labelled data are limited [12].

Interpretability constitutes an additional requirement for clinically oriented image‐analysis systems. High classification performance alone does not establish that a network bases its decisions on diagnostically meaningful retinal structures. Post hoc visualisation techniques such as gradient‐weighted class activation mapping (Grad‐CAM) can provide spatial evidence regarding the image regions that contribute most strongly to a prediction and have been applied to OCT classification to examine the anatomical plausibility of learned responses [13]. Such visualisations should, however, be regarded as supportive interpretability evidence rather than as proof of causal clinical reasoning.

Against this background, the present study addresses the need for an OCT classification framework that combines fine‐grained multi‐disease discrimination, computational efficiency, controlled robustness assessment and visual interpretability. We propose AESNet‐SEC, an attention‐enhanced lightweight deep learning framework that integrates multi‐scale dilated feature extraction, spatial attention, SE channel recalibration and contrastive representation learning. These mechanisms are designed to perform complementary functions: multi‐scale dilated convolutions enlarge the effective receptive field without requiring proportionally deeper convolutional stacks; spatial attention emphasises diagnostically informative image regions; SE blocks adaptively recalibrate channel‐wise responses; and contrastive learning promotes more discriminative feature organisation between disease categories.

The primary aim of this study is to determine whether the integration of these complementary mechanisms can improve multi‐class OCT classification while maintaining computational efficiency. We hypothesised that AESNet‐SEC would provide improved discrimination among retinal disease categories relative to its reduced variants while preserving stable performance under controlled image degradation and when evaluated on independently collected clinical OCT images. The contribution of the principal architectural and training components is therefore examined through explicit ablation analysis rather than inferred solely from the performance of the complete model. For clarity, the model variants are denoted according to the components incorporated into the core architecture: AESNet‐C includes contrastive pretraining, whereas AESNet‐SE incorporates SE recalibration. The complete configuration integrating both components is consistently referred to as AESNet‐SEC throughout the manuscript.

For validation, we utilised two datasets: a public benchmark of 24,000 OCT images spanning eight retinal categories and a prospectively collected clinical dataset comprising 9732 OCT B‐scans from Zeyaei Hospital in Ardakan, Yazd, Iran. The OCT‐C8 benchmark comprises AMD, CNV, CSR, DME, DR, Drusen, macular hole (MH) and Normal images. The clinical cohort contains CNV, DME, Drusen and Normal scans and provides a complementary assessment under routine clinical acquisition conditions. Because the latter originates from a single clinical centre and represents four of the eight benchmark categories, it is used to assess clinical transferability within the evaluated setting rather than to claim multi‐centre generalisability. The study makes three principal contributions:

1. A lightweight multi‐component architecture for eight‐class OCT classification. AESNet‐SEC combines multi‐scale dilated convolutions, spatial attention, SE‐based channel recalibration and contrastive representation learning within a unified framework designed to preserve discriminative capacity while limiting unnecessary computational burden.
2. Component‐resolved evaluation of performance and robustness. The contributions of the principal model configurations are examined through ablation analysis, class‐wise evaluation, ROC analysis, controlled Gaussian‐noise testing and computational‐time assessment, providing a more explicit characterisation of how the complete AESNet‐SEC configuration differs from its reduced variants.
3. Complementary clinical evaluation with visual interpretability. In addition to evaluation on the OCT‐C8 benchmark, the framework is assessed on prospectively collected clinical OCT images. Grad‐CAM visualisations are used to examine whether influential image regions correspond to anatomically plausible retinal abnormalities, with retinal‐specialist review providing qualitative clinical assessment of these activation patterns.

The remainder of this paper is organised as follows. Section 2 reviews related work on deep learning for retinal disease classification and OCT analysis. Section 3 describes the datasets, preprocessing procedures, AESNet‐SEC architecture, training strategy, contrastive‐learning framework and interpretability methodology. Section 4 presents the experimental results, including class‐wise performance, ablation analysis, ROC evaluation, computational time, robustness assessment and clinical evaluation. Section 5 discusses the principal findings, interpretability, comparative performance and study limitations. Section 6 summarises the study and outlines directions for further validation and development.

## 2 Related Work

Deep learning has substantially advanced automated retinal image analysis, particularly for OCT, where disease‐related structural abnormalities can be represented through hierarchical spatial features. Earlier studies primarily investigated transfer learning, conventional CNNs and disease‐specific classification, whereas subsequent work increasingly incorporated multi‐scale representations, attention mechanisms, feature optimisation, denoising and explainability. Despite these advances, substantial heterogeneity remains in the number of diagnostic categories, dataset composition, evaluation protocols, computational requirements and the extent of clinical validation, making direct comparison across studies inherently difficult.

Shah et al. [14] investigated transfer learning for distinguishing Stargardt disease from normal OCT scans and reported 99.6% classification accuracy. Although the study demonstrated that pretrained CNN representations can be effective for OCT analysis, its binary diagnostic setting does not reflect the complexity of simultaneous multi‐disease discrimination. Barua et al. [15] subsequently proposed a multilevel deep‐feature generation framework based on multiple pretrained CNNs and reported accuracies of 97.4% and 100% across two OCT datasets. Shih and Patel [16] also demonstrated the effectiveness of transfer learning and augmentation for four‐class retinal OCT classification, reporting an accuracy of 99.48%. Collectively, these studies established the effectiveness of deep feature extraction for OCT classification, while also illustrating that high performance is often reported under relatively restricted disease settings or dataset‐specific evaluation protocols.

The transition toward broader multi‐class classification has highlighted the need for more discriminative representations. Vasanthi et al. [17] evaluated ManualNet, AlexNet and ResNet across eight macular disease categories, with ResNet providing the strongest performance at approximately 90% accuracy. G et al. [18] compared CNN, VGG‐19 and DenseNet‐121 architectures for multi‐class retinal OCT classification and reported variability among architectures despite explicit attention to class balancing. Das et al. [19] approached limited‐data OCT classification through a data‐efficient semi‐supervised framework incorporating generative adversarial learning rather than relying exclusively on a conventional supervised CNN. These findings indicate that expanding the diagnostic label space changes the learning problem substantially: Architectural depth alone does not guarantee reliable discrimination when disease categories share fine‐grained morphological characteristics.

Attention‐based and multi‐scale strategies have therefore received increasing interest. Rabbi et al. [20] developed a multi‐branch CNN incorporating a convolutional block attention module (CBAM) and reported approximately 96% accuracy in retinal disease classification. The architecture demonstrated the potential value of combining multiple feature pathways with attention‐based refinement, although the evaluated diagnostic space remained smaller than the eight‐category setting considered in the present study. Ebrahimi et al. [21] investigated layer fusion for DR classification using OCT angiography (OCTA), achieving 92.65% accuracy and demonstrating the potential of complementary retinal representations. Because OCTA provides vascular information distinct from structural OCT, however, the imaging setting is not directly equivalent to B‐scan OCT classification.

Other studies have addressed disease‐specific feature extraction and data efficiency. Mukherjee et al. [22] developed a three‐dimensional deep convolutional regression network for retinal‐layer segmentation in patients with AMD, demonstrating accurate delineation of retinal structures. Yin et al. [23] investigated meta‐learning using MAML and MAML++ for OCT pathology classification and reported approximately 95.6% accuracy, highlighting the potential of meta‐learning when labelled samples are limited. Kim and Tran [24] evaluated deep learning models for CNV, DME, Drusen and Normal OCT classification, whereas Vasquez et al. [25] explored interactive deep learning to improve both retinal disease classification and interpretability. The broader review by Badar et al. [26] similarly identified dataset availability, architectural selection and clinically meaningful interpretation as persistent challenges in retinal image analysis.

Robustness to image degradation has also motivated architectures that explicitly integrate denoising and multi‐scale feature extraction. Peng et al. [27] proposed a multi‐scale denoising residual convolutional network for OCT disease classification and reported 96.5% accuracy on OCT‐C4. Khan et al. [28] combined modified DenseNet‐201, InceptionV3 and ResNet‐50 feature extractors with ant colony optimisation and conventional classifiers, reporting 99.1% accuracy with feature optimisation. Moradi et al. [29] integrated retinal‐layer segmentation with ensemble learning for non‐advanced AMD classification and reported an AUC of 99.4%, while also evaluating the method on internal and external data. These studies demonstrate that feature refinement and optimisation can improve disease discrimination, but they also introduce additional design choices whose individual contribution should be distinguished from the performance of the complete pipeline.

More recent work has increasingly emphasised clinically interpretable or disease‐specific analysis. Elkholy and Marzouk [30] investigated CNN‐based four‐class OCT classification involving Normal, DME, CNV and AMD. Hamid et al. [31] combined MobileNetV1‐derived deep features with an LSTM classifier for three‐class AMD classification and reported 98.85% accuracy, 99.09% sensitivity and 99.10% specificity. Hasan et al. [32] developed an explainable framework for OCT‐based glaucoma diagnosis and staging, reporting AUCs of 0.96, 0.98, and 1.00 for early, moderate and advanced glaucoma, respectively, while using feature‐attribution techniques to examine model decisions. Beuse et al. [33] evaluated explainable deep learning for central retinal artery occlusion using OCT data from two German medical centres and nested fivefold cross‐validation, reporting one‐vs‐rest AUCs of 0.96, 0.99 and 0.90 across the evaluated categories. Together, these studies reflect a broader shift from reporting classification accuracy alone toward evaluation strategies that also consider explainability, cross‐validation, disease severity and clinical data heterogeneity. A comparative summary of the representative retinal OCT studies discussed above, including their methodological approaches, diagnostic settings, reported performance and principal scope, is provided in Table 1.

**TABLE 1:** Overview of representative retinal OCT classification and analysis studies.

Ref. (Year)	Method	Diagnostic setting	Reported performance	Relevance and scope
Shah et al. [14]	Pretrained CNN	Stargardt disease vs. Normal	Accuracy: 99.6%	High binary performance; limited diagnostic scope
Barua et al. [15]	Multilevel deep‐feature generation	Retinal OCT abnormalities	Accuracy: 97.4% and 100% across two datasets	Multiple pretrained representations; dataset‐dependent evaluation
Shih and Patel [16]	VGG16 with transfer learning and augmentation	Four OCT categories	Accuracy: 99.48%	Strong four‐class performance
Vasanthi et al. [17]	ManualNet, AlexNet, ResNet	Eight macular categories	Best accuracy: approximately 90%	Broader class space; lower performance than narrower‐class studies
G et al. [18]	CNN, VGG‐19, DenseNet‐121	Multi‐class retinal OCT	Best balanced accuracy: 92.89%	Demonstrates architecture‐dependent variability
Das et al. [19]	GAN‐assisted data‐efficient learning	Four‐class OCT	>10% improvement reported under limited‐data settings	Addresses labelled‐data scarcity
Rabbi et al. [20]	Multi‐branch CNN with CBAM	Retinal OCT classification	Accuracy: 96%	Combines branch‐specific features with attention
Peng et al. [27]	Multi‐scale denoising residual CNN	OCT2017/OCT‐C4	Accuracy: 96.5% on OCT‐C4	Incorporates explicit noise‐oriented feature refinement
Khan et al. [28]	Deep features + ant colony optimisation	Multi‐class OCT	Accuracy: 99.1% with optimisation	Feature optimisation improves discrimination but increases pipeline complexity
Moradi et al. [29]	Segmentation + ensemble learning	Non‐advanced AMD	AUC: 99.4%	Includes internal and external evaluation
Hamid et al. [31]	MobileNetV1 + LSTM	Three AMD classes	Accuracy: 98.85%	Disease‐specific high‐performance classification
Hasan et al. [32]	Explainable machine learning	Glaucoma staging	AUC: 0.96–1.00	Emphasises explainability and disease‐stage discrimination
Beuse et al. [33]	Explainable deep learning	CRAO and differential diagnoses	AUC: 0.90–0.99	Two‐centre dataset and nested five‐fold cross‐validation

Beyond retinal OCT studies, recent methodological work provides relevant evidence for several design decisions that arise in AESNet‐SEC. Hyperparameter selection is particularly important because performance gains cannot be attributed confidently to an architecture when training configurations are insufficiently controlled. Li et al. [34] introduced the multi‐strategy parrot optimiser for medical‐image classification and explicitly performed component‐level ablation, demonstrating that optimisation strategy can materially affect accuracy, precision, recall and F1‐score. Nabeel et al. [35] likewise showed that systematic tuning of model parameters substantially altered classification performance in a medical diagnostic setting. These findings support the need to justify key AESNet‐SEC settings empirically rather than treating dilation rates, dropout, embedding dimensionality, contrastive margin and related training parameters as arbitrary fixed choices.

The combination of multi‐scale receptive fields and attention also has methodological precedent beyond retinal imaging. Yang et al. [36] developed a Multiscale Dilated Adaptive DenseNet with an attention mechanism, demonstrating the utility of combining dilated representations and attention‐based refinement in a complex signal‐classification task. Although the application involved EEG rather than OCT, the architectural principle is relevant to feature extraction across multiple effective receptive fields. More directly within ophthalmic imaging, Yang et al. [37] combined Inception‐V4 with dynamic snow leopard optimisation for DR detection, illustrating how architecture design and explicit optimisation can be jointly considered in retinal disease analysis.

Collectively, the literature reveals several recurring limitations. First, many high‐performing OCT systems are developed for binary, three‐class or four‐class settings, whereas broader disease spaces introduce more difficult inter‐class boundaries. Second, multi‐component architectures frequently combine transfer learning, attention, feature optimisation, augmentation or multi‐scale processing, but the contribution of each element is not always isolated through component‐level evaluation. Third, performance obtained from a single public benchmark does not by itself establish transportability to independently acquired clinical images. Finally, interpretability is increasingly incorporated into retinal deep learning, but visual explanations require cautious interpretation and are strengthened when examined alongside clinically meaningful anatomical findings.

Previous evidence indicates that backbone selection, augmentation and imbalance‐aware learning can materially influence model behaviour under heterogeneous data distributions [38, 39, 40]. Accordingly, AESNet‐SEC was developed under controlled data‐processing and training conditions to reduce confounding effects and more clearly assess the contribution of its feature‐learning components. AESNet‐SEC is positioned within these gaps by combining multi‐scale dilated feature extraction, spatial attention, SE recalibration and contrastive representation learning within a lightweight OCT classification framework. Rather than attributing performance to the complete architecture alone, the evaluation distinguishes reduced AESNet (attention‐enhanced SqueezeNet) configurations from the full AESNet‐SEC model and examines class‐wise discrimination, ROC behaviour, controlled noise robustness, computational time and performance on a prospectively collected clinical OCT cohort. Grad‐CAM analysis further provides visual evidence regarding the retinal regions contributing to model predictions. This combination enables the proposed framework to be assessed not only in terms of aggregate classification performance, but also with respect to component contribution, robustness, computational behaviour and clinically interpretable feature localisation.

## 3 Materials and Methods

The proposed framework was developed through a sequential pipeline comprising image preprocessing, dynamic data processing, feature extraction, contrastive representation learning, supervised optimisation and final classification. Image preprocessing was used to standardise heterogeneous OCT scans before model training, whereas dynamic data processing was introduced to increase training diversity and improve robustness to variations in image appearance. The resulting images were then processed by the proposed AESNet architecture, which combines multi‐scale feature extraction with attention‐based refinement. Contrastive learning was incorporated during pretraining to improve the separability of visually similar retinal disease classes, followed by supervised fine‐tuning for the final classification task. The overall workflow is illustrated in Figure 1.

**FIGURE 1:**
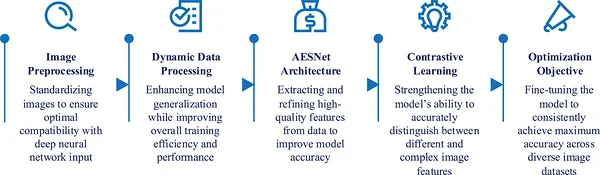
Schematic illustration of the proposed methodology, including image preprocessing, dynamic data processing, AESNet‐based feature extraction, contrastive representation learning and supervised optimisation.

### 3.1 Datasets

The Retinal OCT‐C8 dataset [41] was used as the primary benchmark for multi‐class retinal disease classification. The dataset contains 24,000 OCT images evenly distributed across eight diagnostic categories: Normal, MH, Drusen, DR, DME, Central Serous Retinopathy (CSR), CNV and AMD. The dataset is organised into training, validation and test subsets, providing a balanced experimental setting for model development and evaluation. Representative images from the eight categories are shown in Figure 2. A second dataset was prospectively collected between May 2023 and March 2024 at the Zeyaei Hospital Ophthalmology Clinic in Ardakan, Yazd, Iran, during routine diagnostic and screening procedures. This clinical dataset comprises 9732 high‐resolution OCT B‐scan images belonging to four diagnostic categories: CNV (3147 images), DME (2271 images), Drusen (1184 images) and Normal (3130 images). Each image was independently reviewed and annotated by two experienced board‐certified ophthalmologists, with final confirmation by a senior retina specialist to strengthen diagnostic reliability.

**FIGURE 2:**
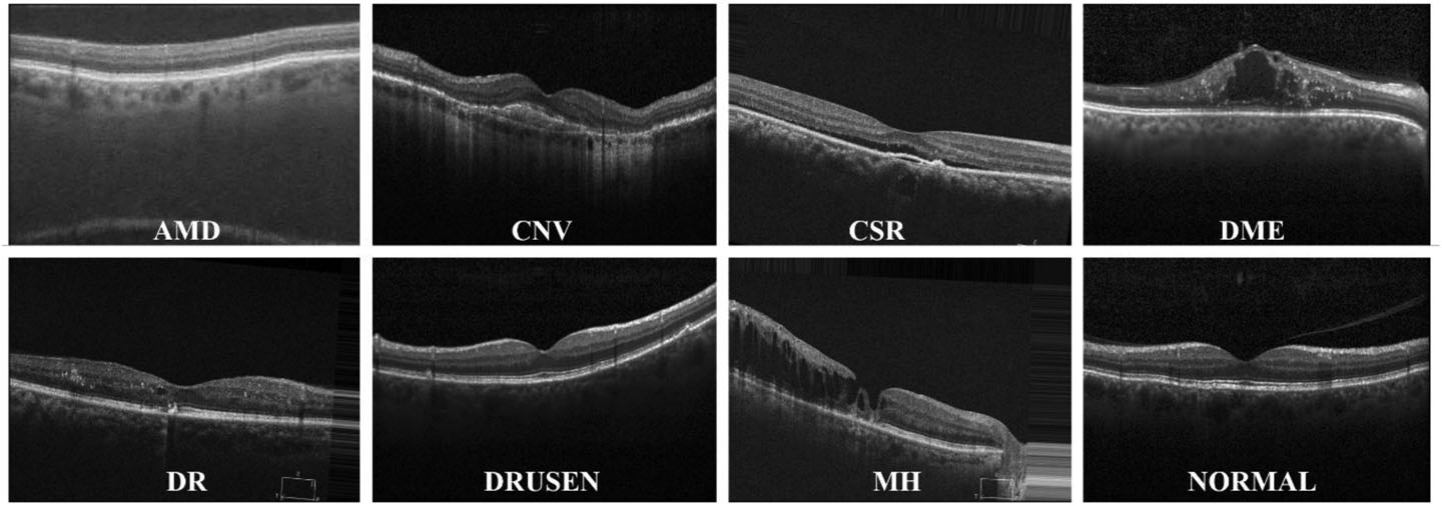
Representative OCT B‐scan images illustrating the eight diagnostic categories in the OCT‐C8 dataset: age‐related macular degeneration (AMD), choroidal neovascularisation (CNV), central serous retinopathy (CSR), diabetic macular edema (DME), diabetic retinopathy (DR), Drusen, macular hole (MH) and Normal.

Unlike the balanced OCT‐C8 benchmark, the clinical dataset retains substantial variation in class frequency and image appearance. It also reflects routine outpatient imaging conditions, including variations in acquisition angle, image contrast and minor motion artefacts. The inclusion of this dataset therefore provides a complementary setting for examining whether the model maintains its discriminative performance beyond a controlled public benchmark.

The clinical data were collected in accordance with the applicable ethical requirements, with patient confidentiality maintained throughout the study and informed consent obtained from all participants. No diagnostic conclusions derived from model predictions were used to establish the reference labels; the ground‐truth annotations were determined independently by the clinical experts. This separation was maintained to prevent model predictions from influencing the clinical reference standard.

### 3.2 Image Preparation and Dynamic Data Processing

To ensure consistency across OCT scans with heterogeneous spatial resolutions, all images underwent a standardised preprocessing procedure involving cropping and resizing. Cropping was guided by the location of the first non‐zero pixel to remove non‐informative peripheral background while retaining the retinal region required for diagnostic interpretation. The cropped images were subsequently resized to a common input dimension to ensure compatibility with the deep learning architecture and to reduce unnecessary computational variation during training.

The preprocessing procedure was deliberately restricted to operations that preserve the underlying retinal morphology. No lesion‐specific manual cropping, retinal‐layer editing or pathology‐guided region selection was introduced before classification. This design reduces the possibility that disease‐specific information is inadvertently embedded into the preprocessing stage.

Dynamic data processing was subsequently applied to increase the diversity of the training images and improve the model's tolerance to moderate acquisition‐related variations. Geometric transformations included horizontal flipping, rotation and scaling, while photometric transformations focused primarily on variations in image intensity and contrast. These transformations were selected because they modify image appearance without intentionally altering the retinal structures that determine the diagnostic label. Excessive geometric deformation was avoided because pronounced transformations could distort clinically meaningful layer geometry or lesion morphology.

Each OCT image was treated as an individual observation and assigned to a single diagnostic category for the classification task. Data augmentation was used to increase representation diversity rather than to create new diagnostic labels. Controlled resampling was additionally used where required to reduce the influence of class‐frequency differences during model development. Gaussian perturbation was also incorporated into the generation of augmented views during contrastive pretraining, as described in Section 3.3.2.

### 3.3 Proposed AESNet‐SEC Framework

The proposed AESNet‐SEC framework was designed to combine computationally efficient feature extraction with spatially and channel‐wise adaptive feature refinement. As illustrated in Figure 3, the architecture consists of a lightweight convolutional backbone, channel shuffling, the proposed AESNet attention block, dropout regularisation and a SE module. Contrastive learning is incorporated at the representation‐learning stage before supervised fine‐tuning. For consistency throughout the manuscript, AESNet denotes the core attention‐enhanced architecture. AESNet‐C refers to AESNet with contrastive pretraining, AESNet‐SE denotes AESNet augmented with SE recalibration, and AESNet‐SEC represents the complete configuration integrating both SE recalibration and contrastive learning. This notation is used consistently in the subsequent ablation experiments.

**FIGURE 3:**
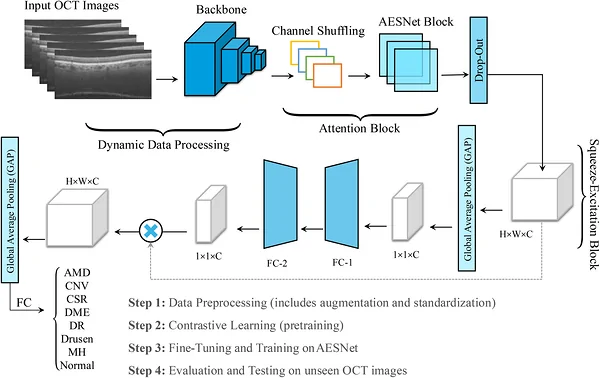
Overview of the proposed AESNet‐SEC framework for retinal disease classification, showing the convolutional backbone, channel‐shuffling operation, AESNet attention block, squeeze‐and‐excitation module, contrastive pretraining stage and final classification pathway.

The backbone produces a feature tensor $F_{m}\in R^{H\times W\times C}$, where C,H and W denote the number of channels, height and width of the feature maps, respectively. The CNN backbone leverages transfer learning to provide an informative initial feature representation while preserving the lightweight design required for efficient OCT classification. The feature maps are subsequently processed by the proposed attention mechanism, which is designed to emphasise discriminative retinal patterns while suppressing less informative responses. A dropout layer with a rate of 0.30 follows the attention block to reduce feature co‐adaptation and limit overfitting. The moderate dropout probability was chosen to provide regularisation without excessively suppressing weak pathological features, which may be diagnostically important in OCT images.

The refined representation is then passed to the SE module, which adaptively recalibrates channel responses according to their relative contribution to the current feature representation. Global average pooling is applied after feature refinement, followed by a fully connected classification layer. Their complementary effects are examined through the reduced AESNet configurations and the complete AESNet‐SEC model, allowing the contribution of the evaluated enhancements to be assessed without relying solely on the performance of the full configuration.

#### 3.3.1 AESNet Unit

Standard convolutions employ a fixed receptive field and may therefore provide limited flexibility when pathological patterns occur at substantially different spatial scales. This issue is particularly relevant in retinal OCT imaging, where abnormalities may range from localised disruptions to broader changes in retinal morphology. Multi‐scale receptive‐field modelling has consequently been adopted in a range of convolutional architectures [42, 43]. Dilated convolution provides an efficient mechanism for enlarging the effective receptive field without requiring proportionally larger convolutional kernels or a corresponding increase in the number of trainable parameters. The AESNet unit therefore employs three parallel dilated‐convolution branches, as illustrated in Figure 4. Before entering these branches, the feature maps undergo channel shuffling to promote information exchange between channels and reduce isolated feature‐group processing.

**FIGURE 4:**
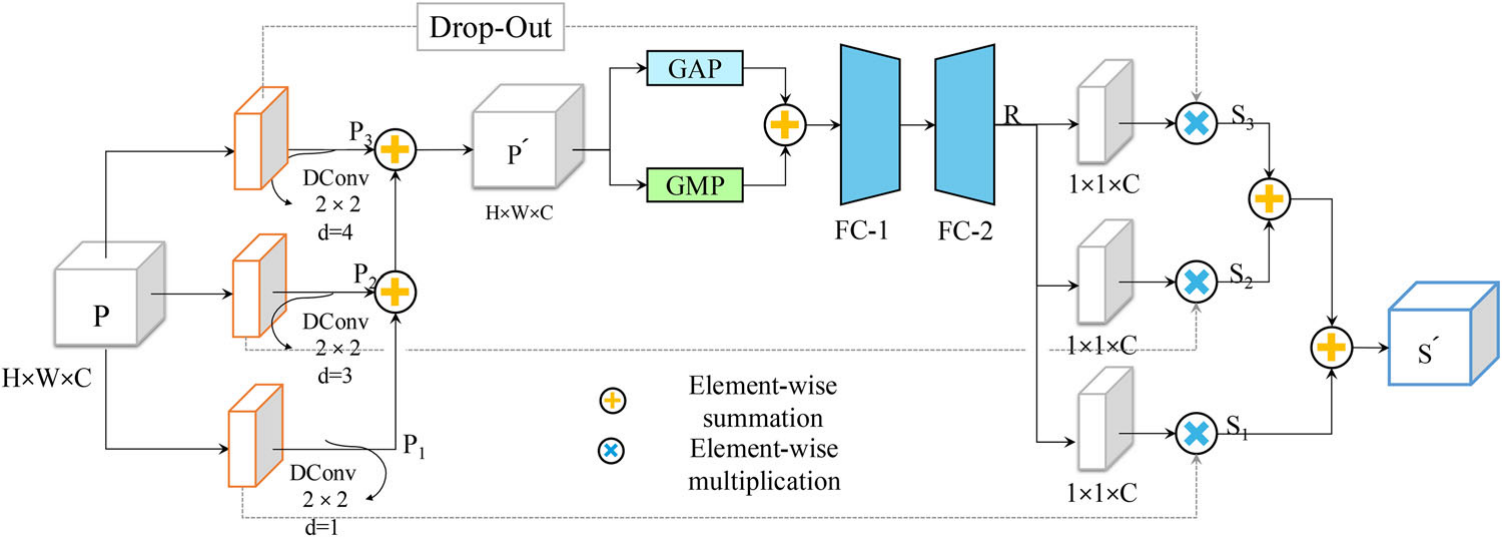
Architecture of the AESNet unit, illustrating parallel dilated‐convolution branches, global average and max pooling, feature recalibration and element‐wise fusion for multi‐scale retinal feature representation.

For an input feature tensor P, the output of the i‐th dilated‐convolution branch is expressed as:

(1) $$\;P_{i}=f_{\mathrm{ReLU}}\;\left[f_{\mathrm{BN}}\left(\mathrm{DCon}v_{d_{i}}\left(P\right)\right)\right],\;i\in \left\{1,2,3\right\}$$

where $d_{i}$ denotes the dilation rate of the corresponding branch, $f_{\mathrm{BN}}$ represents batch normalisation and $f_{\mathrm{ReLU}\;}$ denotes the ReLU activation function. The dilation rates d={1,3,4} were selected to provide complementary local, intermediate and broader receptive‐field coverage while limiting excessively sparse sampling at larger dilation factors. Specifically, the standard branch (d=1) preserves fine retinal details, whereas the d=3 and d=4 branches progressively enlarge the effective receptive field to capture broader disease‐associated structural patterns without requiring substantially larger convolutional kernels. This configuration follows established multi‐scale dilated‐convolution principles [42, 43] and enables complementary spatial contexts to be encoded using the same 2 × 2 kernel size. The outputs of the parallel branches are then fused by element‐wise summation:

(2) $$\;P^{\prime }=P_{1}\;⊕P_{2}⊕P_{3}$$

where ⊕ denotes element‐wise addition. To derive a compact global descriptor, both global max pooling and global average pooling are applied to $P^{\prime }$:

(3) $$Q=f_{\mathrm{GMP}}\;\left(P^{\prime }\right)⊕f_{\mathrm{GAP}}\left(P^{\prime }\right)$$

The two pooling operations provide complementary information: Global average pooling captures the distributed activation pattern across the feature map, whereas global max pooling preserves strongly activated responses that may correspond to localised abnormalities. The resulting representation is regularised using a feature‐drop operation with a dropout probability of 0.25:

(4) $$\;Q^{\prime }=f_{\mathrm{Drop},\;0.25}\;\left(Q\right)$$

This internal dropout operates on the compressed attention representation and is distinct from the 0.30 dropout applied after the main AESNet attention block. The lower rate was used to regularise channel recalibration while preserving sufficient information for subsequent feature weighting. The compressed representation is subsequently transformed through fully connected layers and a sigmoid activation:

(5) $$R=f_{\mathrm{Sig}}\;\left[f_{\mathrm{FC}}\left(Q^{\prime }\right)\right]$$

The resulting weight vector R is multiplied element‐wise by the corresponding multi‐scale features:

(6) $$\;S_{i}=R⊗P_{i},\;i\in \left\{1,2,3\right\}$$

where ⊗ denotes element‐wise multiplication. The recalibrated branches are then combined to produce the final attention‐enhanced representation:

(7) $$\;S^{\prime }=S_{1}\;⊕S_{2}⊕S_{3}$$

#### 3.3.2 Contrastive Learning for Feature Enhancement

Contrastive learning was incorporated into the pretraining stage to improve the structure of the learned feature space. The principal objective is to reduce the distance between alternative views of the same image while increasing separation between images from different diagnostic categories. This strategy is particularly relevant to OCT classification because several retinal diseases share partially overlapping morphological patterns.

Positive pairs were generated by applying stochastic transformations to the same OCT image, including horizontal flipping, rotation, scaling and Gaussian noise. The transformations used for positive‐pair generation were selected to preserve the diagnostic identity of the original scan while introducing sufficient appearance variation to discourage the encoder from relying on transformation‐sensitive image characteristics. Negative pairs were generated by sampling images from different disease categories within the same training mini‐batch. For an input image $x_{i}$, the encoder f(·) generates the latent representation:

(8) $$\;z_{i}=f\left(x_{i}\right)\in R^{d}$$

where d is the embedding dimension. Euclidean distance is used to quantify the separation between a pair of embeddings. The contrastive objective is formulated as:

(9) $$\;L_{\mathrm{contrastive}\;}=\frac{1}{N}\;\sum_{\left(i,j\right)=1}^{N}\left[y_{ij}d{\left(z_{i},z_{j}\right)}^{2}+\left(1-y_{ij}\right)\max{\left(0,m-d\left(z_{i},z_{j}\right)\right)}^{2}\right]$$

where $y_{ij}=1$ for a positive pair and $y_{ij}=0$ for a negative pair, m denotes the contrastive margin and N represents the number of image pairs. An embedding dimension of 128 was used to provide a compact latent representation while retaining sufficient capacity to separate the eight diagnostic categories.

Increasing the embedding dimensionality beyond 128 would increase computational and memory demands, whereas a substantially smaller latent space could constrain the representational capacity required to distinguish retinal diseases with subtle morphological differences. A contrastive margin of 1.0 was adopted to promote sufficient separation between negative pairs without enforcing unnecessarily large inter‐sample distances in the embedding space. The contrastive‐learning parameters were selected to balance representation capacity, inter‐class separation and training stability, while the principal optimisation settings were kept fixed across comparative experiments to minimize configuration‐related variability. The complete hyperparameter configuration used for contrastive pretraining is summarised in Table 2.

**TABLE 2:** Hyperparameter configuration for contrastive pretraining.

Parameter	Value	Description
Embedding dimension d	128	Compact latent representation for inter‐class discrimination
Margin m	1.0	Minimum separation imposed on negative pairs
Distance metric	Euclidean distance	Distance measure between latent embeddings
Positive‐pair strategy	Augmented views of the same image	Flipping, rotation, scaling and Gaussian perturbation
Negative‐pair strategy	Random inter‐class sampling	Images sampled from different disease classes within a mini‐batch
Batch size	64	Number of samples processed in each pretraining batch
Pretraining epochs	50	Duration of contrastive representation learning
Optimiser	Adam	Optimisation algorithm used during pretraining
Learning rate	$1\times 10^{-4}$	Learning rate used for Adam‐based contrastive pretraining
Weight initialisation	Xavier uniform	Initialisation of trainable network parameters

The encoder was pretrained using the contrastive objective for 50 epochs, after which the learned weights were transferred to the classification framework for supervised fine‐tuning with categorical cross‐entropy (CCE). This two‐stage training strategy was designed to organise the latent feature space before supervised optimisation of the final decision boundaries, thereby promoting separation between diagnostically distinct classes while preserving similarity among samples from the same class. Such representation‐level structuring is particularly relevant for retinal conditions with partially overlapping OCT morphologies, including CSR and DME.

#### 3.3.3 Supervised Optimisation Objective

The primary OCT‐C8 experiment is formulated as an eight‐class classification problem in which each OCT image is associated with one mutually exclusive diagnostic category. The final classification layer therefore uses a SoftMax activation function to generate a normalised probability distribution across the eight candidate classes. The model is optimised using CCE:

(10) $$\;L_{\mathrm{CCE}}=-\sum_{i=1}^{C}y_{i}\log\left({\hat{y}}_{i}\right)$$

where C denotes the number of diagnostic classes, $y_{i}$ represents the ground‐truth indicator for class i and ${\hat{y}}_{i}$ is the corresponding predicted probability. For OCT‐C8, C=8. For evaluation on the four‐category clinical dataset, the classification output is adapted to the available CNV, DME, Drusen and Normal categories while retaining the same feature‐learning framework. The probabilistic formulation permits class‐specific evaluation and facilitates subsequent assessment of classification confidence and model interpretability.

### 3.4 Visual Interpretability Assessment

Grad‐CAM was used as a post hoc interpretability method to examine the retinal regions contributing most strongly to AESNet‐SEC predictions. Class‐specific activation maps were generated from the trained network and superimposed on the corresponding OCT images, allowing the spatial distribution of discriminative responses to be visually compared with recognisable retinal abnormalities.

Representative Grad‐CAM outputs were examined for Drusen, DME, CNV and CSR, including disease classes characterised by localised deposits, fluid accumulation or structural alterations of the retinal layers. Two board‐certified ophthalmologists independently reviewed the generated visualisations to determine whether the highly activated regions were anatomically and diagnostically consistent with the corresponding retinal findings. This review was performed only after model inference and did not alter model parameters, reference labels or predictions.

## 4 Results

To assess the diagnostic performance and robustness of the proposed AESNet‐SEC framework, we conducted a comprehensive evaluation using both a balanced benchmark dataset and a real‐world clinical dataset. The model was tested across multiple experimental configurations incorporating data augmentation, contrastive learning and architectural enhancements. Performance metrics including accuracy, sensitivity, specificity, precision and F1‐score were calculated across the eight retinal disease classes to provide a comprehensive assessment. In addition to the overall and class‐wise classification results, the experiments examined convergence behaviour, the individual contributions of the principal architectural and learning components, class‐wise discriminative performance using receiver operating characteristic (ROC) analysis, computational efficiency and stability on prospectively collected clinical OCT images. The following subsections report the quantitative performance and comparative behaviour of AESNet‐SEC across these evaluation settings.

### 4.1 Implementation Details

The implementation of the proposed AESNet model was conducted on a high‐performance computing environment equipped with an NVIDIA RTX 3090 GPU, 32 GB of RAM and an Intel i9 processor. The training and evaluation processes utilised Python and TensorFlow frameworks to ensure efficient deep learning operations. The experimental analysis comprised the primary eight‐class OCT‐C8 classification task, controlled evaluations of the individual AESNet components and a complementary four‐class clinical evaluation using prospectively collected OCT images.

The proposed AESNet architecture integrates advanced mechanisms for efficient and accurate multi‐class classification. At its core, AESNet employs SE blocks to dynamically recalibrate channel‐wise feature importance and attention mechanisms to focus on discriminative regions in OCT images. These features are processed through a lightweight architecture inspired by SqueezeNet, while dilated convolutions capture multi‐scale contextual information that may be useful for recognising retinal abnormalities of different spatial extents.

For model training, the optimisation settings were selected to provide stable convergence while limiting overfitting across the evaluated configurations. The Adam optimiser was used with a learning rate of 1 × $10^{-4}$, whereas experiments employing SGD used a learning rate of $5\times 10^{-4}$; a decay factor of $1\times 10^{-6}$ was applied to support gradual optimisation. A batch size of 32 was adopted for the principal supervised experiments, with a batch size of 16 used in selected configurations. Regularisation was implemented through dropout with a rate of 0.30 and early stopping with a patience of 10 epochs to reduce overfitting and improve training stability.

The OCT‐C8 experiments followed the 60%/20%/20% allocation used in the reported five‐fold evaluation, corresponding to 14,400 training images, 4800 validation images and 4800 test images. Within each class, 1800 images were assigned to training and 600 each to validation and testing. Patient‐level identifiers were not available in the retained OCT‐C8 metadata; consequently, partitioning was performed at the image level, and strict patient‐level independence across the training, validation and test sets could not be independently verified. Accordingly, repeated or adjacent B‐scans originating from the same subject, if present in the source dataset, could not be explicitly grouped during partitioning. Data augmentation was applied exclusively to the training partition, increasing the effective training set from 14,400 to 20,000 images; validation and test images were retained without augmentation. Augmentation was performed only after partition assignment, preventing augmented derivatives of evaluation images from entering the training process. The distribution is summarised in Table 3.

**TABLE 3:** Distribution of the OCT‐C8 dataset across the eight retinal disease classes, including the original training allocation, augmented training size, validation set and test set.

Class	Samples	Training	After augmentation	Validation	Testing
Age‐related macular degeneration (AMD)	3000	1800	2500	600	600
Choroidal neovascularisation (CNV)	3000	1800	2500	600	600
Central serous retinopathy (CSR)	3000	1800	2500	600	600
Diabetic macular edema (DME)	3000	1800	2500	600	600
Diabetic retinopathy (DR)	3000	1800	2500	600	600
Drusen	3000	1800	2500	600	600
Macular hole (MH)	3000	1800	2500	600	600
Normal	3000	1800	2500	600	600
Total	24,000	14,400	20,000	4800	4800

Advanced preprocessing techniques were also employed to standardise input images. Cropping, horizontal flipping, geometric transformations and image enhancement were used as described in Section 3. Because OCT‐C8 is balanced at the class level, augmentation was used primarily to increase within‐class appearance diversity rather than to alter the underlying class priors. The reported five‐fold evaluation used systematic rotation of the data partitions to reduce dependence on a single split and to examine the stability of training and validation behaviour across different partitions.

Statistical comparisons among the AESNet configurations were conducted over multiple repeated evaluations performed under matched experimental conditions. Each modified configuration was compared with the baseline AESNet using a two‐sided Wilcoxon signed‐rank test, with Holm adjustment applied to the three pairwise comparisons to control for multiple testing. Statistical significance was defined at an adjusted p<0.05, and accuracy uncertainty was additionally characterised using 95% confidence intervals.

### 4.2 Classification Performance

The automated model for detecting retinal diseases from OCT images demonstrated strong class‐wise performance, as summarised in Table 4. For AMD, the model achieved a sensitivity of 94.72% and an accuracy of 99.08%, indicating effective recognition of AMD while maintaining a low overall misclassification rate. For CNV, the corresponding sensitivity and accuracy were 93.99% and 98.94%, respectively. To avoid ambiguity associated with fractional confusion counts obtained from aggregated evaluations, the class‐wise results are reported directly in terms of the derived diagnostic metrics. These metrics provide a more interpretable comparison of performance across the eight disease categories.

**TABLE 4:** Class‐wise performance of the proposed retinal disease classification framework on OCT‐C8.

Class	Sensitivity (%)	Accuracy (%)	Specificity (%)	Precision (%)	F1‐score (%)
AMD	94.72	99.08	99.64	97.65	96.17
CNV	93.99	98.94	99.61	97.39	95.67
CSR	91.27	98.56	99.50	96.59	93.87
DME	92.05	98.64	99.64	96.80	94.40
DR	88.93	97.90	99.33	95.36	92.07
Drusen	88.33	97.78	99.23	94.76	91.45
MH	97.49	99.51	99.85	98.92	98.20
Normal	96.60	99.41	99.80	98.57	97.58
Average	92.44	98.78	99.58	97.10	94.56

The average sensitivity of 92.44% indicates strong disease recognition across the eight categories, whereas the average accuracy of 98.78% reflects a low overall misclassification rate. The mean specificity of 99.58% indicates effective rejection of non‐target classes, while the precision of 97.10% and F1‐score of 94.56% demonstrate a favourable balance between positive predictive performance and sensitivity.

Despite the strong overall results, disease‐specific differences were evident. DR and Drusen exhibited the lowest sensitivities, at 88.93% and 88.33%, respectively. These results suggest that the principal residual errors are concentrated in classes for which OCT manifestations may be relatively subtle or overlap with structural features observed in other retinal conditions. Importantly, the high corresponding specificities of 99.33% and 99.23% indicate that the reduced sensitivity in these classes was not accompanied by a substantial increase in false‐positive classification. For CSR, the model achieved 91.27% sensitivity, 98.56% accuracy and an F1‐score of 93.87%. The lower sensitivity relative to MH, Normal, AMD and CNV is consistent with the greater visual overlap that can arise between fluid‐associated retinal abnormalities such as CSR and DME. In contrast, MH exhibited the highest sensitivity at 97.49% and an accuracy of 99.51%, while Normal images were identified with 96.60% sensitivity and 99.41% accuracy. Figure 5 presents representative confusion matrices for the four AESNet configurations on a common 4800‐image held‐out OCT‐C8 test partition. The training and validation behaviour of AESNet‐SEC across the repeated five‐fold evaluations is illustrated in Figure 6.

**FIGURE 5:**
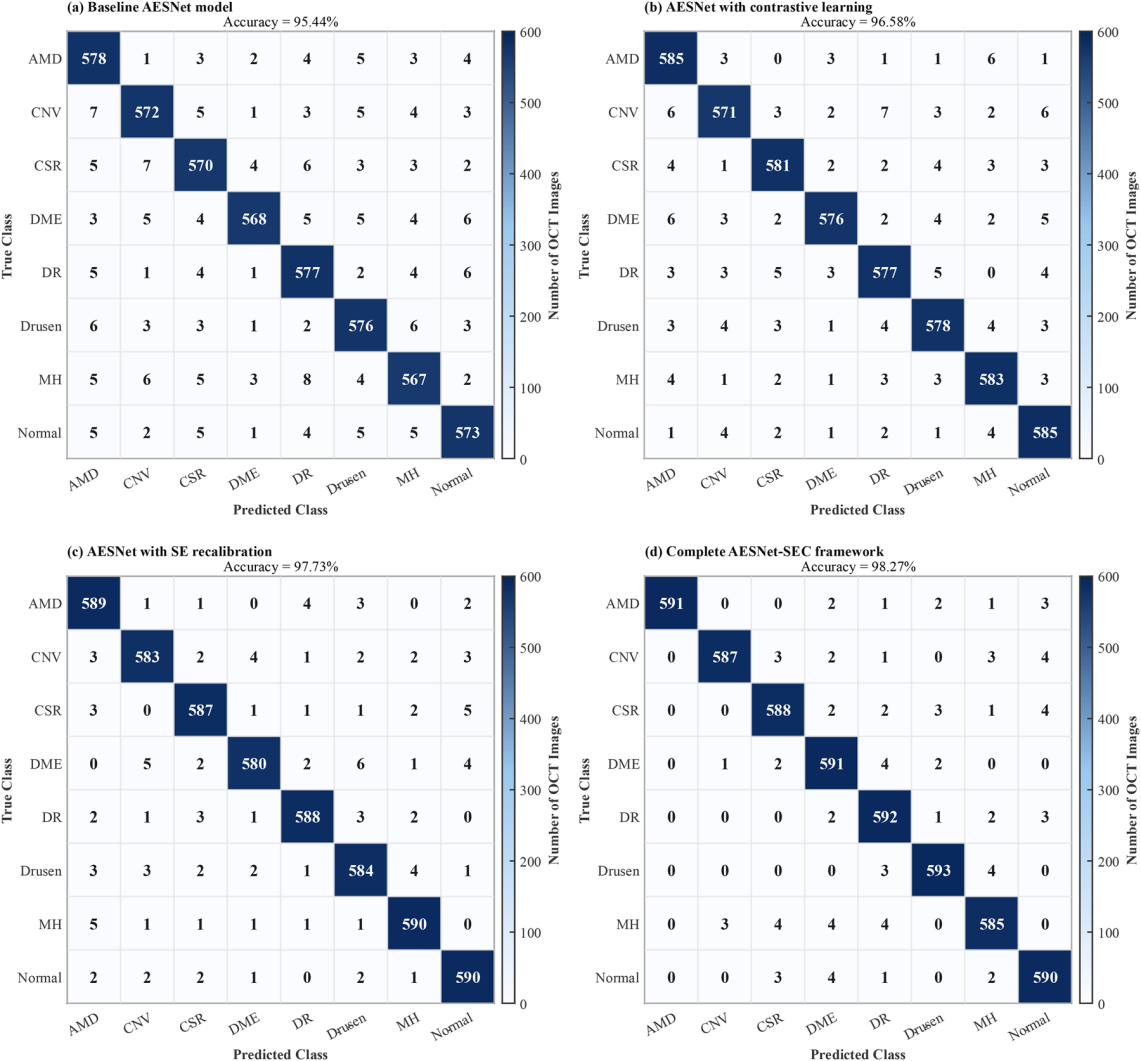
Confusion matrices of the AESNet variants on the OCT‐C8 test set: (a) baseline AESNet, (b) AESNet with contrastive learning, (c) AESNet with squeeze‐and‐excitation recalibration and (d) the complete AESNet‐SEC framework.

**FIGURE 6:**
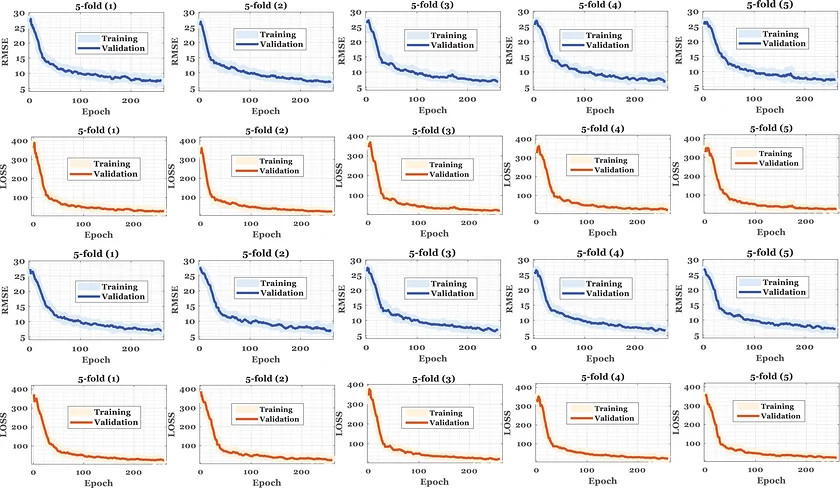
Performance metrics (RMSE and loss) during training and validation across two sets of five‐fold cross‐validation for the AESNet‐SEC model. This figure illustrates the model's performance in terms of RMSE and loss during training and validation across five different folds.

Because these matrices correspond to a specific evaluation realisation, their confusion‐matrix‐derived accuracies are not expected to be numerically identical to the repeated‐evaluation summary values reported in Table 5. The confusion matrices therefore provide a class‐resolved view of the changes summarised by the aggregate performance metrics and show that the performance improvement is distributed across several disease categories rather than arising from a single class. The RMSE and loss curves shown in Figure 6 further characterise optimisation behaviour across two repeated five‐fold evaluation sets. In each fold, training and validation RMSE decrease markedly during the early epochs and subsequently approach a relatively stable region. The corresponding loss curves exhibit a similar pattern, with rapid initial reduction followed by gradual convergence.

**TABLE 5:** Performance comparison of baseline architectures and AESNet variants based on the repeated evaluation protocol used for component‐level ablation.

Model	Accuracy (%)	Sensitivity (%)	Specificity (%)	Precision (%)	F1‐score (%)
VGG19	94.85	91.20	96.50	92.35	91.77
DenseNet	96.45	93.80	97.80	94.75	94.27
ResNet	96.85	94.50	97.95	95.20	94.85
AESNet	97.28	94.72	99.64	97.65	96.17
AESNet‐C	97.54	93.99	99.61	97.39	95.67
AESNet‐SE	98.25	92.05	99.64	96.80	94.40
AESNet‐SEC	98.56	97.49	99.85	98.92	98.20

The close trajectories of the training and validation curves indicate that optimisation remained stable across the evaluated partitions, with no persistent late‐stage divergence between the two curves. Although convergence behaviour alone does not establish external generalisability, the recurrence of similar trends across folds provides evidence that the reported training dynamics were not driven by a single favourable partition.

### 4.3 Ablation Study

Table 5 provides a detailed comparison of seven models, including the baseline architectures VGG19, DenseNet and ResNet, together with AESNet and its progressively modified variants AESNet‐C, AESNet‐SE and AESNet‐SEC. The complete AESNet‐SEC configuration achieved the highest reported accuracy of 98.56%, together with 97.49% sensitivity, 99.85% specificity, 98.92% precision and an F1‐score of 98.20%.

The values reported in Table 5 summarise the component‐level performance obtained under the repeated evaluation protocol and therefore represent the primary ablation comparison. By contrast, the confusion matrices in Figure 5 correspond to a representative 4800‐image held‐out test realisation, from which the confusion‐matrix‐derived statistics in Table 6 were calculated. Relative to AESNet, the complete AESNet‐SEC configuration increased accuracy by 1.28 percentage points, sensitivity by 2.77 points, specificity by 0.21 points, precision by 1.27 points and F1‐score by 2.03 points. Importantly, the intermediate variants did not improve every metric monotonically. AESNet‐C increased accuracy from 97.28% to 97.54% but showed modest reductions in sensitivity and F1‐score, while AESNet‐SE increased accuracy to 98.25% without producing a corresponding increase in sensitivity. These non‐monotonic changes indicate that contrastive representation learning and SE‐based channel recalibration influence different aspects of the classification behaviour, whereas their integration in AESNet‐SEC produced the strongest overall balance across the reported metrics. The ablation analysis was structured at two complementary levels. The architectural comparison in Table 5 isolates the incremental effects of SE recalibration and contrastive learning within the AESNet framework, whereas the ROC analysis in Section 4.4 separately evaluates the contributions of augmentation and contrastive learning to class‐wise discrimination.

**TABLE 6:** Statistical and performance comparison of AESNet variants on the OCT‐C8 test set.

Metric	AESNet	AESNet‐C	AESNet‐SE	AESNet‐SEC
Correct classifications/4800	4581	4636	4691	4717
Accuracy (%)	95.44	96.58	97.73	98.27
95% CI for accuracy (%)	94.81–95.99	96.03–97.06	97.27–98.11	97.86–98.60
Macro sensitivity (%)	95.44	96.58	97.73	98.27
Macro specificity (%)	99.35	99.51	99.68	99.75
Macro precision (%)	95.45	96.59	97.73	98.28
Macro F1‐score (%)	95.44	96.58	97.73	98.27
Accuracy gain vs. AESNet (percentage points)	0.00	+1.15	+2.29	+2.83
Relative error reduction vs. AESNet (%)	0.00	25.11	50.23	62.10
Statistical significance vs. AESNet across 100 repeated runs	—	*p* < 0.01	*p* < 0.01	*p* < 0.01

To complement the component‐wise ablation results with an explicit assessment of uncertainty and statistical robustness, the four AESNet configurations were further compared using confusion‐matrix‐derived performance measures, 95% confidence intervals and repeated‐run statistical analysis. This analysis was designed to determine whether the observed improvements were consistent beyond differences in point accuracy alone. The resulting performance and uncertainty measures are summarised in Table 6.

As shown in Table 6, performance improved progressively from the baseline AESNet to the complete AESNet‐SEC configuration. AESNet‐SEC achieved the highest accuracy of 98.27% (95% CI: 97.86–98.60), representing a 2.83‐percentage‐point gain over AESNet and reducing the classification error from 4.56% to 1.73%, equivalent to a 62.10% relative error reduction. The intermediate AESNet‐C and AESNet‐SE configurations also yielded statistically significant improvements relative to the baseline, while the complete AESNet‐SEC configuration exhibited the strongest overall performance; the repeated‐run comparisons remained significant at *p* < 0.01. Collectively, these findings provide complementary evidence that the observed performance gains are consistent across the evaluated configurations rather than being reflected solely by a single aggregate accuracy value.

Figure 7 reports empirical computational time for VGG19, DenseNet, ResNet, AESNet‐C, AESNet‐SE and AESNet‐SEC across five randomly selected test groups. The measured values remained approximately within the 0.62–0.75 s range for all evaluated configurations. Because these measurements depend on the GPU, implementation, test‐group size and software environment, they characterise the observed computational time under the specified experimental conditions rather than a device‐independent measure of computational complexity. For a convolutional layer, the computational complexity can be expressed as:

(11) $$O\left(\;\mathrm{complexity}\;\right)=k^{2}\;C_{\mathrm{in}\;}C_{\mathrm{out}\;}HW$$

where k is the kernel size; $C_{\mathrm{in}}$ and $C_{\mathrm{out}}$ denote the numbers of input and output channels, respectively; and H and W represent the spatial dimensions of the feature map. For AESNet‐SEC, the attention and SE components introduce additional operations; however, the architecture is built on a lightweight feature‐extraction framework.

**FIGURE 7:**
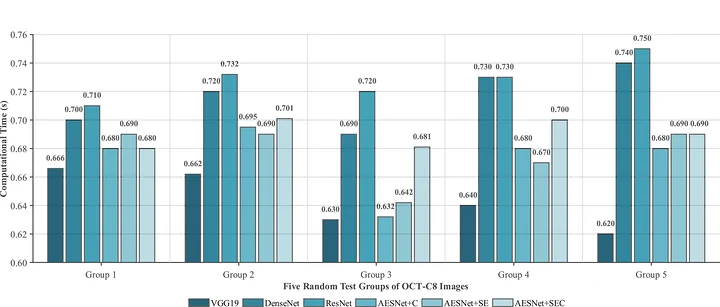
Empirical computational time of VGG19, DenseNet, ResNet, AESNet‐C, AESNet‐SE and AESNet‐SEC across five randomly selected OCT‐C8 test groups.

The empirical timing results in Figure 7 indicate that these additions did not produce a substantial inference‐time penalty under the evaluated hardware configuration. The observed sub‐second processing time supports computational feasibility for rapid OCT analysis, although it should not by itself be interpreted as proof of real‐time clinical deployment on all hardware platforms.

### 4.4 Impact of Visual Features, Augmentation and Contrastive Learning

One of the principal challenges in multi‐disease OCT classification is maintaining high sensitivity while preserving specificity among diseases that may share partially overlapping structural characteristics. Disease‐specific morphology therefore influences the difficulty of the classification problem. MH, CNV and AMD exhibit relatively distinctive structural patterns in OCT images. In contrast, CSR and DME may share fluid‐associated changes within or beneath the retinal layers, increasing the potential for inter‐class confusion. Normal OCT scans provide a comparatively stable reference because they lack the pathological structural abnormalities present in the disease categories.

Figure 8 presents ROC curves for all eight classes under four controlled learning scenarios: (1) neither augmentation nor contrastive learning, (2) augmentation without contrastive learning, (3) contrastive learning without augmentation and (4) the combination of augmentation and contrastive learning. This experiment provides a complementary learning‐component ablation that is independent of the architectural comparison in Table 5.

**FIGURE 8:**
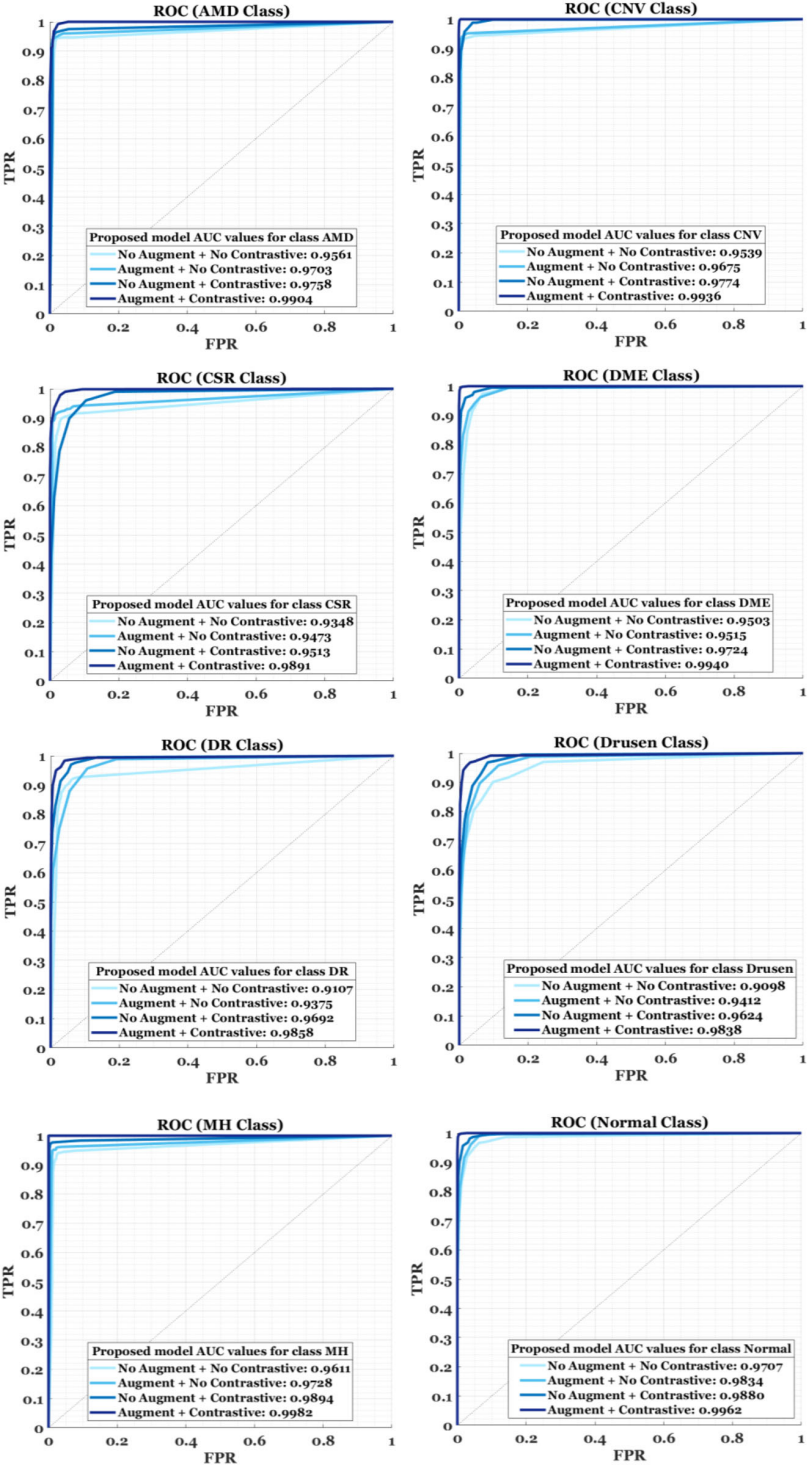
ROC curves and class‐wise AUC values for the eight retinal disease categories under four learning configurations: no augmentation/no contrastive learning, augmentation only, contrastive learning only and combined augmentation and contrastive learning.

For AMD, the AUC increased from 0.9561 without augmentation or contrastive learning to 0.9703 with augmentation alone and 0.9758 with contrastive learning alone, reaching 0.9904 when both were combined. CNV followed a similar progression from 0.9539 to 0.9675, 0.9774 and 0.9936. The same pattern was observed for CSR (0.9348 → 0.9473 → 0.9513 → 0.9891), DME (0.9503 → 0.9515 → 0.9724 → 0.9940), DR (0.9107 → 0.9375 → 0.9692 → 0.9858), Drusen (0.9098 → 0.9412 → 0.9624 → 0.9838), MH (0.9611 → 0.9728 → 0.9894 → 0.9982) and Normal (0.9707 → 0.9834 → 0.9880 → 0.9962).

Across the eight classes, the mean class‐wise AUC increased from 0.9434 in the unaugmented/non‐contrastive setting to 0.9589 with augmentation alone and 0.9732 with contrastive learning alone, reaching 0.9914 when the two strategies were combined. The largest absolute gains between the baseline and combined setting occurred for DR (+0.0751), Drusen (+0.0740) and CSR (+0.0543), which were among the more difficult classes in the sensitivity analysis. This finding indicates that augmentation and contrastive representation learning are particularly beneficial for classes with less distinctive or more overlapping OCT morphology.

MH achieved the highest AUC in the combined configuration (0.9982), followed closely by Normal (0.9962), DME (0.9940) and CNV (0.9936). In sum, the ROC results show that augmentation and contrastive learning provide complementary improvements in class discrimination, with the combined configuration producing the highest AUC for every evaluated disease category.

### 4.5 Clinical Generalisability

To evaluate performance beyond the OCT‐C8 benchmark, the proposed framework was assessed using the prospectively collected clinical dataset of 9732 OCT B‐scans acquired at Zeyaei Hospital Ophthalmology Clinic in Ardakan, Yazd, Iran, between May 2023 and March 2024. The dataset includes CNV (3147 images), DME (2271), Drusen (1184) and Normal (3130) and incorporates routine clinical variability in acquisition angle, contrast and minor motion artefacts.

The clinical cohort was evaluated in four repeated runs. Each confusion matrix in Figure 9 contains the complete 9732‐image clinical cohort rather than a disjoint one‐quarter subset; the four matrices therefore represent repeated evaluations of the same clinical dataset and should not be described as four independent clinical cohorts. The corresponding accuracies were 96.88%, 96.33%, 96.68% and 96.98%.

**FIGURE 9:**
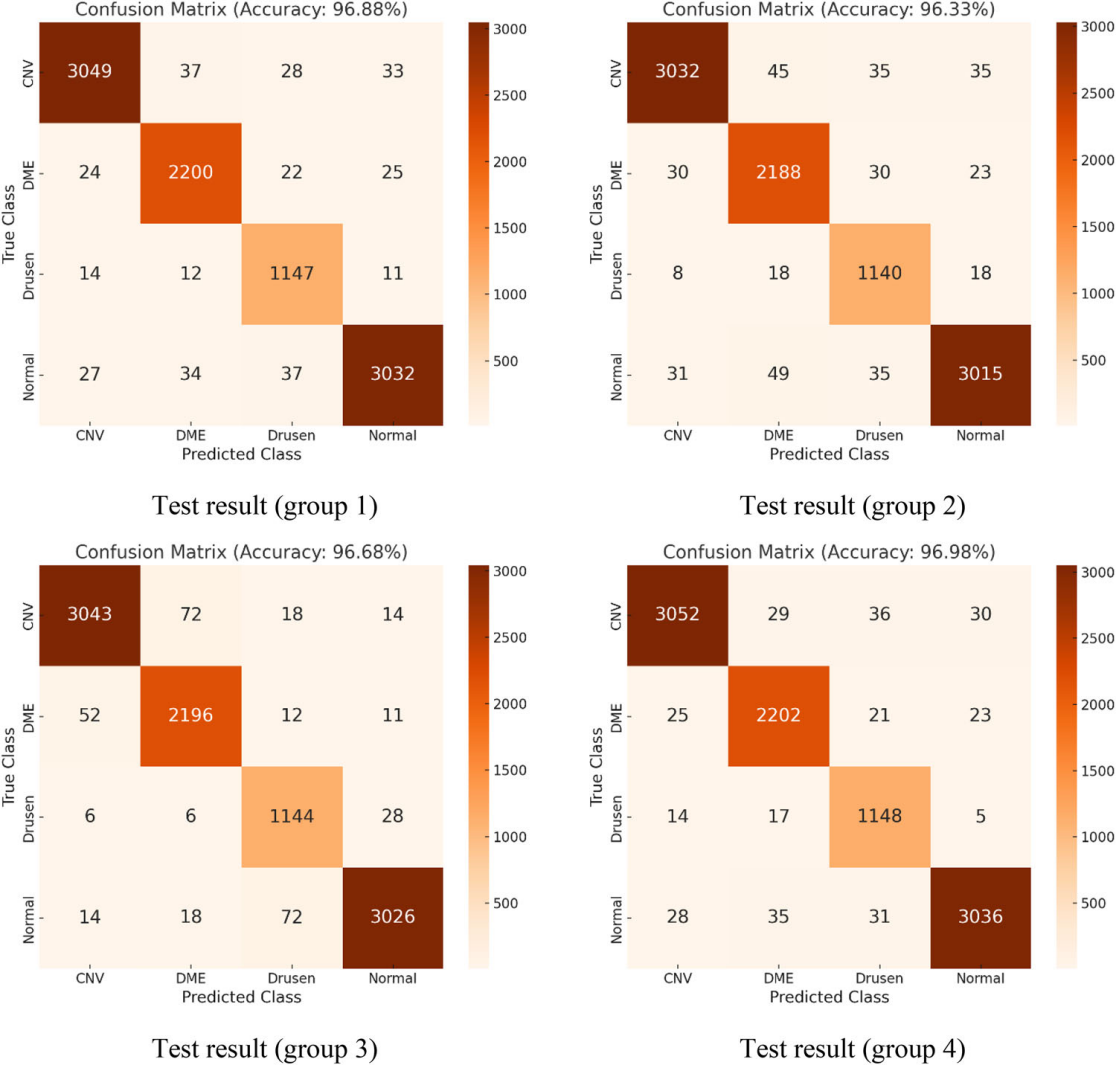
Confusion matrices from four repeated evaluations of the complete 9732‐image clinical OCT cohort, showing consistently high accuracy (96.33%–96.98%) and low misclassification rates across the four evaluation runs.

Across the four runs, mean accuracy was 96.72% ± 0.29% (SD), with an observed range of 96.33%–96.98%. Based on the confusion‐matrix counts, the Wilson 95% confidence intervals for overall accuracy were 96.51%–97.20% for Run 1, 95.94%–96.69% for Run 2, 96.31%–97.02% for Run 3 and 96.62%–97.30% for Run 4. The narrow spread between repeated runs indicates limited run‐to‐run variability on this clinical cohort.

The confusion matrices in Figure 9 show consistently dominant diagonal entries for CNV, DME, Drusen and Normal across all four evaluations. In Run 1, 3049 of 3147 CNV scans, 2200 of 2271 DME scans, 1147 of 1184 Drusen scans and 3032 of 3130 Normal scans were correctly classified. Comparable class‐wise behaviour was retained in the remaining runs, indicating that the variation in overall accuracy was not driven by a single diagnostic category.

The clinical results are particularly relevant because they were obtained from images collected under routine diagnostic conditions rather than from the balanced OCT‐C8 benchmark. Nevertheless, these findings should be interpreted as image‐level external evaluation at a single clinical centre. The present experiment does not, by itself, establish performance across independent institutions, acquisition devices or populations, nor does it establish robustness to severe acquisition artefacts or ungradable OCT scans. Patient‐level identifiers were not retained in the analysis metadata; consequently, the independence of repeated B‐scans from individual subjects could not be verified. The clinical results should therefore be interpreted as image‐level external evaluation rather than as strictly patient‐independent validation.

## 5 Discussion

The proposed AESNet‐SEC framework achieved strong multi‐class OCT classification performance while combining attention‐guided feature refinement, SE recalibration, dilated multi‐scale feature extraction and contrastive representation learning within a lightweight architecture. The complete configuration reached an accuracy of 98.56% on OCT‐C8 and maintained consistently high performance in the prospectively collected clinical cohort. Importantly, these results should be interpreted together with the component‐level ablation, noise‐robustness analysis, repeated clinical evaluations, computational‐time measurements and Grad‐CAM visualisations rather than on the basis of accuracy alone.

The architecture appears particularly effective when disease categories exhibit either distinctive structural abnormalities or partially overlapping retinal morphology. Attention and SE‐based recalibration provide complementary spatial and channel‐wise feature weighting, while contrastive pretraining encourages greater separation between diagnostically different representations. The resulting improvement is therefore most appropriately interpreted as the combined effect of these mechanisms rather than as evidence that any single module independently accounts for the final performance. Moreover, the clinical findings support the potential use of AESNet‐SEC as an OCT‐based decision‐support framework, but they do not by themselves establish readiness for autonomous clinical deployment.

### 5.1 Performance and Robustness Analysis

To assess the model's robustness to controlled image degradation, Gaussian noise (*μ* = 0, *σ* = 0.01) was introduced to the OCT images. As presented in Table 7, AESNet‐SEC preserved relatively narrow class‐level confidence intervals after perturbation, although the intervals shifted downward to varying degrees across disease categories. For example, the interval for AMD changed from [0.95, 0.99] under the baseline condition to [0.92, 0.96] after noise addition, whereas the corresponding interval for Drusen shifted from [0.89, 0.94] to [0.84, 0.89].

**TABLE 7:** Class‐level performance intervals under baseline and Gaussian‐noise conditions, together with independent qualitative assessments by two ophthalmologists.

Class	Baseline CI	CI with noise	Ophthalmologist 1	Ophthalmologist 2
AMD	[0.95, 0.99]	[0.92, 0.96]	Clear retinal alterations were consistently recognised.	Performance remained strong, with a modest reduction under noise.
CNV	[0.94, 0.98]	[0.90, 0.94]	Characteristic abnormalities remained readily identifiable.	Noise had a limited effect on recognition.
CSR	[0.92, 0.97]	[0.88, 0.93]	Greater sensitivity to image degradation was observed.	Early or subtle manifestations remained more challenging.
DME	[0.93, 0.97]	[0.89, 0.93]	Fluid‐related abnormalities remained recognisable.	Performance remained strong, although subtle findings were more affected.
DR	[0.90, 0.95]	[0.85, 0.90]	Subtle structural abnormalities were more sensitive to noise.	Early manifestations remained comparatively difficult to identify.
Drusen	[0.89, 0.94]	[0.84, 0.89]	Small or subtle deposits were more affected by perturbation.	Recognition of early changes remained challenging under noise.
MH	[0.96, 0.99]	[0.93, 0.97]	The characteristic macular defect remained readily recognisable.	Only a limited effect of noise was observed.
Normal	[0.97, 0.99]	[0.95, 0.98]	Normal retinal morphology remained readily distinguishable.	Performance remained stable despite image perturbation.

The class‐dependent response is informative because the largest degradation occurred primarily in disease categories with comparatively subtle OCT manifestations. Drusen and DR showed greater deterioration than MH and Normal, which remained comparatively stable under the same perturbation. This pattern is consistent with the class‐wise results in Section 4, where Drusen and DR also exhibited the lowest sensitivities among the eight categories. Thus, the noise experiment does not simply indicate a uniform decline in performance; rather, it identifies the disease categories for which subtle structural information is most vulnerable to image degradation.

The ophthalmologist assessments were broadly consistent with the quantitative class‐level behaviour. Disease categories characterised by conspicuous structural changes, including MH and AMD, remained comparatively stable, whereas DR and Drusen were considered more sensitive to degradation because their diagnostic manifestations can be less pronounced. This qualitative agreement supports the clinical plausibility of the observed error pattern but should not be interpreted as a formal statistical agreement analysis between the model and clinicians.

The ablation results provide additional insight into the role of the individual design components. Relative to AESNet, which achieved 97.28% accuracy, the addition of contrastive learning increased accuracy to 97.54%, whereas SE‐based recalibration increased it to 98.25%. The complete AESNet‐SEC configuration reached 98.56%, corresponding to a 1.28‐percentage‐point improvement over AESNet. More importantly, the complete model increased sensitivity from 94.72% to 97.49% and F1‐score from 96.17% to 98.20%. The intermediate configurations did not improve every metric monotonically; AESNet‐C and AESNet‐SE increased accuracy while showing lower sensitivity than the complete configuration. This non‐monotonic behaviour indicates that the individual components affect different aspects of the decision boundary and that their principal benefit emerges when spatial attention, channel recalibration and contrastive representation learning are combined.

The ROC analysis supports the same interpretation from a class‐discrimination perspective. The combined augmentation–contrastive configuration produced the highest AUC for every disease category, with the mean class‐wise AUC increasing from 0.9434 without either strategy to 0.9914 when both were applied. The largest improvements were observed for DR, Drusen and CSR, again suggesting that representation enhancement is particularly beneficial for classes with less distinctive or partially overlapping OCT morphology.

The repeated clinical evaluations provide a complementary assessment of stability outside the balanced OCT‐C8 benchmark. Accuracy across the four evaluations ranged from 96.33% to 96.98%, with a mean of 96.72% and limited run‐to‐run variability. These repeated evaluations should, however, be interpreted as repeated assessments of the same 9732‐image clinical cohort rather than as four independent external cohorts. Consequently, they demonstrate numerical stability within the evaluated clinical dataset but do not establish reproducibility across independent institutions or patient populations.

The Gaussian‐noise experiment likewise represents a controlled stress test rather than a complete surrogate for clinical image degradation. Real‐world OCT artefacts may include severe motion, shadowing, segmentation‐related distortions, signal attenuation, defocus, device‐specific acquisition characteristics or partially ungradable scans. Performance under such conditions cannot be inferred directly from the low‐amplitude Gaussian‐noise experiment and requires dedicated evaluation.

### 5.2 Interpretability

To further improve the model's interpretability, we applied Grad‐CAM to visualise which regions of the OCT images most strongly influenced the model's decisions. Figure 10 illustrates representative activation maps for Drusen, DME, CNV and CSR, together with the corresponding OCT scans and activation overlays. The visualisations indicate that high‐response regions generally coincide with retinal structures relevant to the predicted disease category. For Drusen, activation was concentrated around localised subretinal alterations, whereas the DME and CSR examples showed attention over regions associated with fluid‐related structural changes. In the CNV example, the activation map emphasised the abnormal retinal region rather than non‐retinal background. These observations are consistent with the intended function of the attention‐enhanced architecture, which is to prioritise discriminative anatomical information rather than global image appearance alone.

**FIGURE 10:**
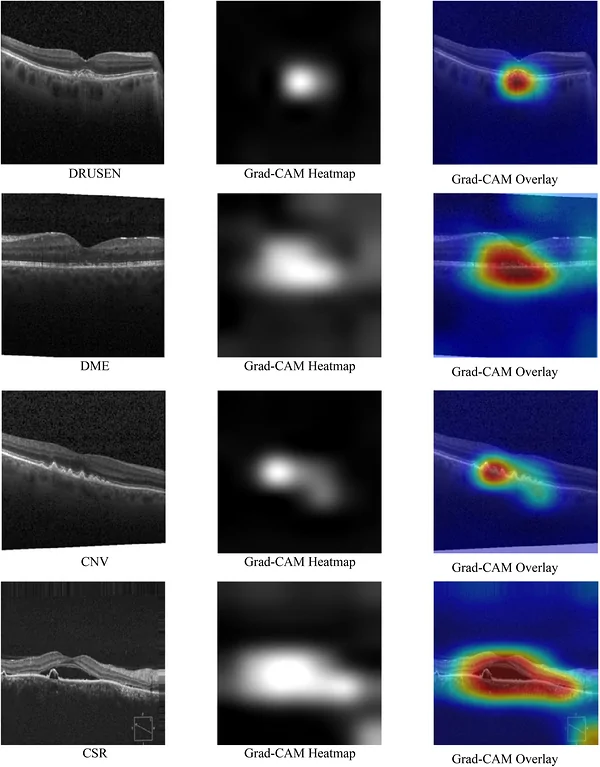
Representative Grad‐CAM visualisations for Drusen, DME, CNV and CSR, showing the original OCT image, class‐specific activation heatmap and corresponding overlay.

Two retinal specialists independently reviewed the Grad‐CAM outputs and considered the highlighted regions to be broadly consistent with expected disease‐related findings. This clinical review provides qualitative support for the anatomical plausibility of the model's attention patterns and addresses an important distinction between high predictive performance and clinically interpretable behaviour.

Nevertheless, Grad‐CAM should be interpreted as a post hoc visualisation of model sensitivity rather than as proof that the highlighted regions are causal biomarkers or that the model reproduces ophthalmologist reasoning. The present analysis did not use lesion‐level reference masks, localisation metrics, or formal inter‐reader agreement statistics. Accordingly, the ophthalmologist review provides qualitative interpretability evidence rather than quantitative validation of lesion localisation. Future work should complement these visualisations with expert‐annotated lesion regions and quantitative localisation measures to determine how consistently model attention overlaps clinically defined abnormalities.

### 5.3 Comparison and Limitations

Direct comparison among published retinal‐classification studies is complicated by differences in dataset composition, disease definitions, class cardinality, preprocessing, train–test partitioning and evaluation metrics. Accordingly, the most informative comparisons in this study are those performed under the common OCT‐C8 experimental protocol. Literature‐reported results obtained on different disease tasks are retained only as contextual reference points and should not be interpreted as strict head‐to‐head benchmarks.

The comparative results in Table 8 show that AESNet‐SEC achieved an accuracy of 98.56%, sensitivity of 97.49% and specificity of 99.85%. Within the OCT‐C8 comparisons reported here, these results indicate a favourable balance between disease sensitivity and rejection of non‐target classes. The advantage is also consistent with the internal ablation results, in which the complete architecture outperformed the baseline AESNet configuration across accuracy, sensitivity, precision and F1‐score.

**TABLE 8:** Comparative performance of AESNet‐SEC and selected retinal image classification methods. Results obtained under different datasets or disease definitions are included for context and are not necessarily directly comparable with OCT‐C8.

Ref. (Year)	Method	Accuracy (%)	Sensitivity (%)	Specificity (%)	Comparison context
Sunija et al. [5]	OctNET (Lightweight CNN)	93.85	91.32	94.78	Lightweight OCT classification framework
Akinniyi et al. [6]	Multi‐scale feature ensemble	94.67	92.15	95.50	Multi‐scale retinal feature representation
Das et al. [19]	GAN‐assisted data‐efficient OCT classification	94.30	91.85	94.88	Data‐efficient learning under limited labelled data
Rabbi et al. [20]	Multi‐branch CNN with CBAM	88.44	86.29	89.70	Attention‐based multi‐branch representation
G et al. [18]	CNN, VGG‐19, DenseNet‐121	87.93	85.50	89.22	Comparison of conventional CNN backbones
Tan et al. [11]	Semi‐supervised transfer learning	89.92	87.85	91.10	Semi‐supervised OCT classification
Peng et al. [27]	Multi‐scale denoising residual CNN	91.25	89.75	92.10	Noise‐oriented multi‐scale feature learning
Saha et al. [8]	Lightweight CNN with global‐local fusion and SE recalibration	96.01	—	—	Recent lightweight OCT classification with channel recalibration
Khriss et al. [9]	AMS‐PSO‐optimised CNN	95.24	—	—	Systematic hyperparameter optimisation with ablation and sensitivity analysis
Ajmal et al. [44]	VGG19 with preprocessing, augmentation and latent‐space SMOTE	97.54	—	—	Clinical OCT classification with independent external validation; external accuracy 96.07%
Qian et al. [45]	IP‐Triplet with DenseNet backbone	98.18	98.18	98.90	OCT‐C8 evaluation using triplet‐based representation learning
Proposed	AESNet‐SEC	98.56	97.49	99.85	Eight‐class OCT‐C8 classification in the present study

Lightweight design should also be considered together with measured computational time rather than inferred solely from architectural labels. Across the five OCT‐C8 test groups evaluated in Section 4, AESNet‐SEC required a mean computational time of approximately 0.690 s, compared with approximately 0.716 s for DenseNet and 0.728 s for ResNet under the same hardware environment. VGG19 remained faster at approximately 0.644 s. Thus, AESNet‐SEC does not provide the minimum measured execution time, but it achieves its higher classification performance without the computational‐time increase observed for the larger DenseNet and ResNet comparisons. Because these values are hardware‐ and implementation‐dependent, they should not be extrapolated directly to other deployment platforms.

Cross‐study comparisons in Table 8 are necessarily contextual because the evaluated studies differ in dataset composition, class cardinality, preprocessing pipelines and validation protocols. Recent OCT investigations further indicate that lightweight architectural design, systematic hyperparameter optimisation and external validation can substantially influence reported performance [8, 9, 44]. Qian et al. [45] reported an accuracy of 98.18% on OCT‐C8 using an IP‐Triplet representation‐learning framework, providing a closely related benchmark for the present eight‐class classification setting. AESNet‐SEC achieved an accuracy of 98.56%, with 97.49% sensitivity and 99.85% specificity. These results position AESNet‐SEC competitively among recent OCT classification approaches, while differences between independently conducted studies remain contingent on their respective experimental conditions and therefore do not constitute direct evidence of statistical superiority.

Several limitations remain. First, although the clinical cohort provides a valuable evaluation beyond the public benchmark, it was collected at a single centre and contains only four of the eight OCT‐C8 categories. The results therefore do not establish generalisability across institutions, OCT devices, acquisition protocols, geographic populations or the full eight‐class disease spectrum. Second, patient‐level identifiers were unavailable in the retained analysis metadata, precluding verification of strict subject‐level independence across repeated B‐scans. If multiple images originated from the same patient or eye, image‐level evaluation may yield more favourable performance than a strictly patient‐disjoint protocol because adjacent or repeated OCT scans can share substantial anatomical information. Future validation should therefore use explicitly patient‐grouped partitions in which all scans from a given subject are confined to a single evaluation subset.

Third, although augmentation was restricted to the training data, synthetic transformations cannot reproduce the full distribution of unseen clinical variation. More severe motion artefacts, signal dropout, poor fixation, low‐quality scans, uncommon disease presentations and coexisting pathologies may affect performance differently from the transformations examined here. A prospective multi‐centre study with patient‐disjoint evaluation and prespecified image‐quality strata would therefore provide a stronger assessment of clinical generalisability.

Moreover, diseases with more subtle structural features, such as DR and Drusen, remain challenging to classify with perfect sensitivity, indicating areas where the model can be refined. This limitation is consistent across the class‐wise sensitivity, ROC and noise‐robustness analyses and suggests that future improvements should prioritise disease categories characterised by small or heterogeneous structural abnormalities rather than focusing exclusively on aggregate accuracy.

Additionally, the reliance on single‐modal OCT data may limit the diagnostic potential of the system, particularly in cases where multimodal imaging, such as OCT combined with fundus photography, could provide complementary information. The present framework also operates on individual B‐scans and therefore does not exploit the complete three‐dimensional structure available in volumetric OCT examinations. Extending the framework to volumetric and multimodal inputs may improve discrimination in cases where a single cross‐sectional image provides incomplete diagnostic information.

Variability in imaging protocols, acquisition characteristics and disease presentation remains a major challenge to the generalisation of OCT‐based models across clinical settings [3]. Although the Grad‐CAM analysis in the present study demonstrates that AESNet‐SEC concentrates on anatomically relevant retinal regions, post hoc saliency provides supportive rather than definitive evidence of clinical interpretability [13]. Future validation should therefore incorporate patient‐disjoint multi‐centre cohorts, heterogeneous acquisition conditions, quantitative localisation assessment and broader retinal imaging information. In this context, OCTA‐derived vascular‐density representations have demonstrated discriminative value for differentiating proliferative from non‐proliferative DR [46], supporting the potential extension of AESNet‐SEC toward complementary structural and vascular retinal information. Such evaluation would provide a more rigorous basis for establishing the robustness and clinical transferability of the framework across heterogeneous ophthalmic workflows.

## 6 Conclusion

The integration of artificial intelligence into ophthalmic diagnostics represents an important direction in digital health, particularly for scalable retinal image analysis in settings where specialist interpretation may be limited. OCT provides detailed structural information on the retina, yet reliable interpretation requires substantial clinical expertise. In this study, we developed AESNet‐SEC, a lightweight deep learning framework for multi‐class retinal disease classification that integrates attention‐guided feature refinement, SE recalibration, dilated multi‐scale feature extraction and contrastive representation learning. AESNet‐SEC achieved strong classification performance on the eight‐class OCT‐C8 benchmark and maintained consistently high accuracy in the prospectively collected four‐class clinical cohort. The ablation and ROC analyses further indicated that the complete configuration provided the most balanced performance among the evaluated AESNet variants, particularly when augmentation and contrastive learning were combined. The framework also showed resilience to controlled Gaussian‐noise perturbation, although the effect of image degradation was more pronounced for classes with subtler retinal manifestations, including DR and Drusen.

Grad‐CAM analysis provided qualitative evidence that the model concentrated on anatomically relevant retinal regions, and independent review by retinal specialists supported the clinical plausibility of the highlighted areas. These findings strengthen the interpretability of the framework but do not substitute for quantitative localisation validation or prospective clinical evaluation. In sum, AESNet‐SEC offers a favourable balance among classification performance, computational efficiency, and visual interpretability for OCT‐based retinal disease analysis. Further validation using patient‐disjoint, multi‐centre cohorts, heterogeneous OCT devices, broader image‐quality conditions and quantitative explainability measures will be necessary before clinical deployment can be established. Future work should also examine volumetric OCT, multimodal retinal imaging and prospective human‐in‐the‐loop evaluation to determine the framework's reliability and practical value across diverse clinical workflows.

## Author Contributions

**Khosro Rezaee**: conceptualisation, methodology, software, formal analysis, writing – original draft, writing – review and editing, visualisation, project administration. **Safoura Farsi Khavari**: methodology, investigation, resources, writing – review and editing. **Hossein Ghayoumi Zadeh**: methodology, data curation, writing – review and editing.

## Conflicts of Interest

The authors declare no conflicts of interest.

## Funding

The authors have nothing to report.

## AI‐Assisted Writing Declaration

The authors affirm that the writing of this manuscript was entirely their own. AI‐based tools were used only to refine grammar and improve clarity, similar to how one might use a language editor. No AI‐generated content contributed to the scientific ideas, analysis or structure of the paper. Every sentence was carefully reviewed and shaped by the authors to ensure the integrity, originality and academic quality of the work. We take full responsibility for all aspects of the manuscript.

## Data Availability

Requests for access to both raw and processed data, excluding programming code, will undergo a thorough review by the relevant institutional and legal authorities to ensure adherence to intellectual property rights and confidentiality agreements. While the Zeyaei Ophthalmology Clinic data from Ardakan, Yazd, Iran, may be considered for sharing, such requests will be contingent upon a valid, well‐justified need and will be subject to appropriate ethical and legal approval processes. Any data shared will be governed by a material transfer agreement to protect the interests of all parties involved. Access to patient‐related information will be provided only if a compelling justification is presented, ensuring that patient privacy and confidentiality are rigorously maintained. In addition, the Kaggle dataset used for model training is available upon request. Programming code will be accessible in accordance with the terms of the GNU General Public License version 3.
